# Guessing right – whether and how medical students give incorrect reasons for their correct diagnoses

**DOI:** 10.3205/zma001293

**Published:** 2019-11-15

**Authors:** Leah T. Braun, Katharina F. Borrmann, Christian Lottspeich, Daniel A. Heinrich, Jan Kiesewetter, Martin R. Fischer, Ralf Schmidmaier

**Affiliations:** 1 Ludwig-Maximilians-University (LMU) Munich, Klinikum der Universität München, Medizinische Klinik und Poliklinik IV, München, Germany; 2 Ludwig-Maximilians-University (LMU) Munich, Klinikum der Universität München, Institut für Didaktik und Ausbildungsforschung in der Medizin, München, Germany

**Keywords:** Clinical reasoning, diagnostic errors, qualitative research, virtual patients

## Abstract

**Background: **Clinical reasoning is one of the central competencies in everyday clinical practice. Diagnostic competence is often measured based on diagnostic accuracy. It is implicitly assumed that a correct diagnosis is based on a proper diagnostic process, although this has never been empirically tested. The frequency and nature of errors in students’ diagnostic processes in correctly solved cases was analyzed in this study.

**Method: **148 medical students processed 15 virtual patient cases in internal medicine. After each case, they were asked to state their final diagnosis and justify it. These explanations were qualitatively analyzed and assigned to one of the following three categories:

1. correct explanation,
2. incorrect explanation and
3. diagnosis guessed right.

**Results: **The correct diagnosis was made 1,135 times out of 2,080 diagnostic processes. The analysis of the associated diagnostic explanations showed that

1. 92% (1,042) reasoning processes were correct,
2. 7% (80) were incorrect, and
3. 1% (13) of the diagnoses were guessed right.

Causes of incorrect diagnostic processes were primarily a lack of pathophysiological knowledge (50%) and a lack of diagnostic skills (30%).

**Conclusion: **Generally, if the diagnosis is correct, the diagnostic process is also correct. The rate of guessed diagnoses is quite low at 1%. Nevertheless, about every 14th correct diagnosis is based on a false diagnostic explanation and thus, a wrong diagnostic process. To assess the diagnostic competence, both the diagnosis result and the diagnostic process should be recorded.

## Zusammenfassung

**Zielsetzung:** Diagnostizieren ist eine der zentralen Kompetenzen im klinischen Alltag. Die Diagnosekompetenz wird häufig anhand der Diagnoserichtigkeit gemessen. Es wird implizit angenommen, dass einer richtigen Diagnose ein richtiger Diagnoseprozess zugrunde liegt und dass nicht geraten wird, obwohl dies bisher nie empirisch überprüft wurde. Die Häufigkeit und die Art von Fehlern im studentischen Diagnoseprozess bei richtigem Diagnoseergebnis wurden in dieser Studie analysiert.

**Methodik: **148 Medizinstudierende im klinischen Studienabschnitt bearbeiteten 15 virtuelle Patientenfälle. Nach jedem Fall wurden sie aufgefordert, eine finale Diagnose zu stellen und diese zu begründen. Diese Begründungen wurden inhaltsanalytisch ausgewertet und jeweils einer der drei folgenden Kategorien zugeordnet:

1. korrekte Diagnosebegründung,
2. fehlerhafte Begründung der Diagnose und
3. zufällig richtig geratene Diagnosen.

**Ergebnisse:** Bei 2080 ausgewerteten Diagnoseprozessen wurde 1135 Mal die richtige Diagnose gestellt. Die Analyse der dazugehörigen Diagnosebegründungen ergab, dass

1. 92% (1042) Begründungen korrekt waren,
2. 7% (80) fehlerhaft waren und
3. 1% (13) der Diagnosen richtig geraten wurden.

Ursachen für fehlerhafte Diagnoseprozesse waren in erster Linie mangelndes pathophysiologisches Wissen (50%) sowie mangelnde diagnostische Fähigkeiten (30%).

**Schlussfolgerung:** In der Regel ist bei korrekt gestellter Diagnose auch der Diagnoseprozess korrekt. Die Rate an geratenen Diagnosen ist mit 1% sehr gering. Dennoch liegt ca. jeder 14. richtigen Diagnose eine falsche Diagnosebegründung und damit ein falscher Diagnoseprozess zugrunde. Zum Assessment der Diagnosekompetenz sollten daher das Diagnoseergebnis und der Diagnoseprozess erfasst werden.

## Introduction

During an average working day, a general practitioner on average sees 45 patients [1] and makes many diagnostic decisions. The rate for a doctor working in a clinic is probably similar. This illustrates the importance of diagnostic competence in everyday clinical practice. Because of this, diagnostic competence is also one of the central topics in medical education research and part of the medical curriculum.

Diagnostic competence can be captured using several parameters: A standard method is to measure the diagnostic accuracy as a result parameter (often binary coded: right vs wrong) [2], [3], [4]. For example, diagnostic efficiency (number of correctly diagnosed cases divided by the time needed for diagnosis) can be used to record diagnostic process quality [2]. For a meaningful assessment of improved diagnostic knowledge, not only factual knowledge but also conditional and procedural knowledge should be recorded (e.g. 3-component test) [5]. For optimal assessment of diagnostic competence it is helpful to combine different assessment methods, such as the diagnostic result and one of the above-mentioned parameters to measure the diagnostic process – Ilgen [6] emphasizes that diagnostic competence does not end with arriving at a correct diagnosis, but the associated diagnostic process too playing a role. Also, the analysis of the causes of diagnostic errors [7] or the cognitive steps during diagnosis [8] can be used to determine diagnostic competence or deficits in the clinical decision process. The causes leading to misdiagnosis were also investigated for medical students [7] based on Graber’s classification [9]: There are eight different cognitive reasons for incorrect diagnoses: lack of diagnostic skills (for example in the interpretation of an ECG), lack of knowledge, faulty context generation, faulty triggering, misidentification (e.g. myocarditis and endocarditis), premature closure, overestimating and underestimating findings, and failure to find any diagnosis at all. One problem here, however, is that it is not yet known how many of the correct diagnoses were guessed right or whether these correct diagnoses are based on a faulty diagnostic process.

While quite a lot is known about the diagnostic process and the explanations for misdiagnoses comparatively speaking, so far only a few studies have been carried out which look at correct diagnoses. Previously, it was assumed that a correct diagnosis is based on a correct diagnosis process with a correct diagnostic explanation, but this has never been empirically verified to our knowledge.

Against this background, the following research question should be answered: How many correct diagnoses are based on a wrong diagnostic process, and what error types are there? How many of the correct diagnoses were guessed right?

To answer this question, the diagnostic explanations of medical students in a controlled setting were qualitatively evaluated.

## Method

### Study design and participants 

This article presents the qualitative data from a large, randomized intervention study analyzing the effects of various scaffolding methods (representation prompts, structured reflection and feedback) on the diagnostic skills of medical students. The quantitative results of this study are part of another publication [10].

During the summer of 2017, 151 advanced medical students in the clinical study section of the Ludwig Maximilian University and the Technical University of Munich processed 15 virtual patient cases on the electronic learning platform CASUS [11]. All subjects volunteered to participate in the study. Prerequisite for participation was the completion of the internal medicine module (6^th^ and 7^th^ semester). Participants were made aware of the study via circulars and notice boards. The cases were carefully piloted with ten students. Following a socio-demographic questionnaire, a test established the participants’ prior knowledge and an introductory video explained the technicalities of the learning platform. The participants then worked on 15 cases, apart from a control group that solved only 10 cases. After the medical history and the physical examination in each case, participants had access to virtual patient records with data from various technical examinations (such as laboratory results, an ECG or an X-ray). Finally, students had to state and justify their final diagnosis. An exemplary typology of two cases, according to Huwendiek et al. is shown in table 1 (Tab. 1) [12]. Participants received a financial allowance of 30 Euros. For the study, a certificate of compliance was issued by the Ethics Committee of the LMU Munich (number 75-16).

#### Evaluation and statistics

The correct diagnoses were determined in advance by the case authors (LB and KB) and a team of experts consisting of four physicians. Furthermore, it has been determined which information (technical examinations and key terms) had to be included in an explanation in order to classify these as correct.

There was an exploratory examination of all correct diagnoses. After a coding scheme had been developed, all justifications were assigned to one of the following three categories: right reasoning, wrong reasoning and guessed diagnoses. The category “right reasoning” included all explanations in which no wrong statements were made. The “wrong explanation” category included all justifications that contained some objective error, such as a misdiagnosed ECG or an incorrect pathophysiological explanation of symptoms. The category “guessed diagnoses” only included justifications in which the subjects explicitly stated that they had guessed the diagnosis. The definitions of the three categories, as well as suitable examples, are shown in table 2 (Tab. 2).

All wrong explanations were also discussed jointly by two of the authors (LB and RS) and assigned to a further category. The wrong explanations were then subdivided according to which aspects were wrong. The categorization was based on the classification for diagnostic errors by medical students [7]. Four categories were distinguished: lack of diagnostic skills regarding the interpretation of technical examination findings, lack of pathophysiological knowledge, incorrect causal relationships and general uncertainty in the diagnosis.

The statistical evaluation was carried out using SPSS 25.

## Results

### Participants 

148 out of 151 participants processed all cases and were included in the data evaluation. The students on average were 25.3 (SD=3.3) years old and had 3.3 (SD=1.0) months of clinical experience. The final average grade was 1.6 (SD=0.6), the grade in internal medicine was 2.2 (SD=1.3), and the oral and written physics grade was 2.3 (SD=1.0) or 2.5 (SD=0.9).

#### Diagnostic evidence and forms of diagnostic reasoning

In total, over 2,000 diagnostic processes were recorded, of which 814 ended with a misdiagnosis. The correct diagnosis was made in 1,135 diagnostic processes.

Many of the diagnostic explanations (between 86 and 100%) were correct, except for case 7 (heart failure), where only 70% of the explanations were correct. There was no correlation between the overall difficulty of a case – reflected by the diagnostic accuracy – and the rate of erroneous reasoning (see table 3 (Tab. 3)). Almost none of the correct solutions were guessed: the rate of correctly guessed diagnoses was between 0 and 4.5% per case (see table 3 (Tab. 3)). The diagnostic explanations did not improve in thematically similar cases with the same diagnosis.

In all cases, 80 reasons were wrong (7%). These were assigned to the four categories mentioned above. Examples of all categories are shown in table 4 (Tab. 4). Lack of pathophysiological knowledge was the most common reason for errors, with 50% (40 out of 80 errors), followed by a lack of diagnostic skills (30%).

## Discussion

We were able to show in this study that a faulty diagnostic process was behind 7% of correct diagnoses. There are four different causes for these errors: Lack of pathophysiological knowledge, lack of diagnostic skills, incorrect causal relationships and the inability to reduce the diagnostic uncertainty through the diagnostic process.

Considering the results, the following aspects are striking: The number of correctly guessed diagnoses is low, and clearly below the statistically expected rate of probability: apart from cases 10 and 15, the cases all had the main symptom of dyspnoea, for which only a limited number of diagnoses is possible other than exotic diagnoses and atypical outcomes.

Each case was designed to have 3 possible differential diagnoses following examination of the medical history; the additional information (physical examination and technical examination) then in each case pointed towards a specific diagnosis. Therefore, an approximate probability rate of about 30% can be assumed. However, very few students randomly made the right diagnosis. There was a well-thought-out diagnostic process behind almost all mentioned diagnoses. In another study, it was demonstrated regarding incorrect diagnoses that only a small number of diagnoses are due to a complete lack of knowledge [7].

Overall, this is a good result: Diagnoses are not guessed in experimental and virtual settings but are usually based on a well-thought-out – even if incorrect – diagnostic process. 

Comparable to the causes of diagnostic errors, similar sources of errors could also be identified in this study. A lack of knowledge and a lack of diagnostic skills should not be underestimated as a source of errors despite the somewhat contradictory study situation [9], [13] and should be addressed in the medical curriculum. Overall, it has been confirmed that determining the diagnostic process is essential because giving a correct diagnosis does not always imply a faultless diagnostic process. For future studies in the field of clinical reasoning, therefore, both the diagnostic result and the diagnostic process should be recorded in order to gain a comprehensive picture of a person’s diagnostic competence. Computer-aided methods of text analysis could be helpful here.

Despite a large number of over 2,000 diagnostic processes analyzed, the results of this study are limited to the field of internal medicine and should also be replicated with cases from other specializations. Furthermore, we were only about to formulate statements regarding the diagnostic processes of medical students; it is not possible to draw conclusions about other levels of expertise.

It is advantageous that the diagnostic processes were not disturbed by the methodology of our study design – as can be the case with the use of think-aloud-protocols, for example [14]. For teaching purposes, it would be desirable in the future, if in addition to feedback on the case solution in the processing of virtual patient cases, individual feedback on the reasoning could be given.

## Conclusions

In this study, for the first time, the diagnostic explanations of correct diagnoses were analyzed in a controlled setting. 7% of the correct diagnoses are based on erroneous diagnostic processes; 1% of the diagnoses were simply guessed right.

## Competing interests

The authors declare that they have no competing interests. 

## Figures and Tables

**Table 1:**
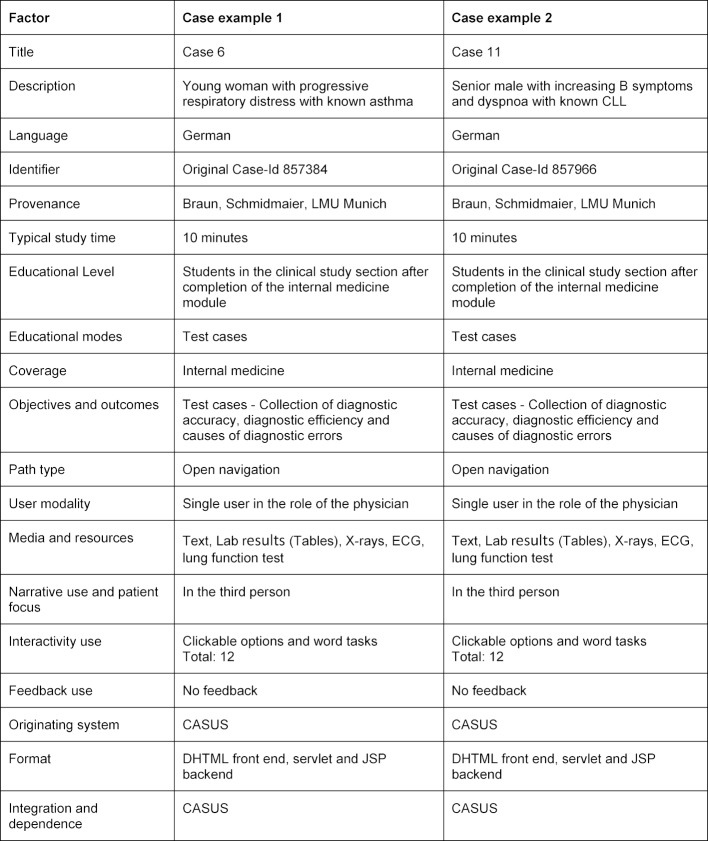
Exemplary typology of a patient case

**Table 2:**
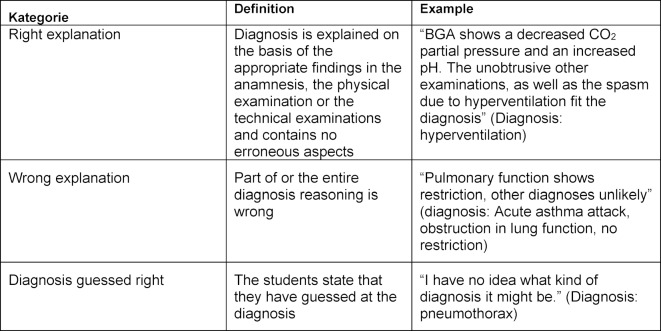
Examples of diagnostic explanations

**Table 3:**
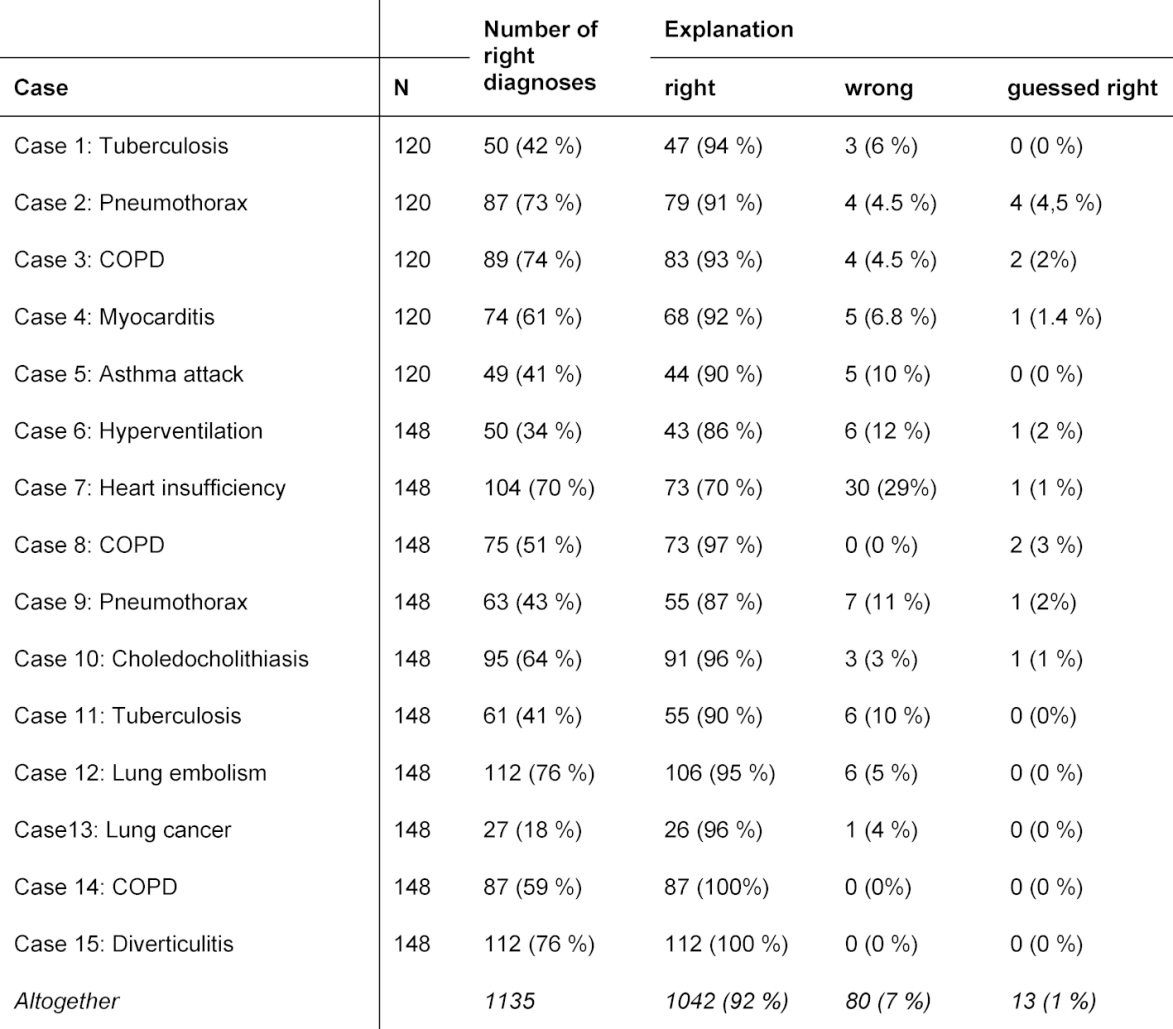
Frequency of right and wrong explanations and guessed diagnosis

**Table 4:**
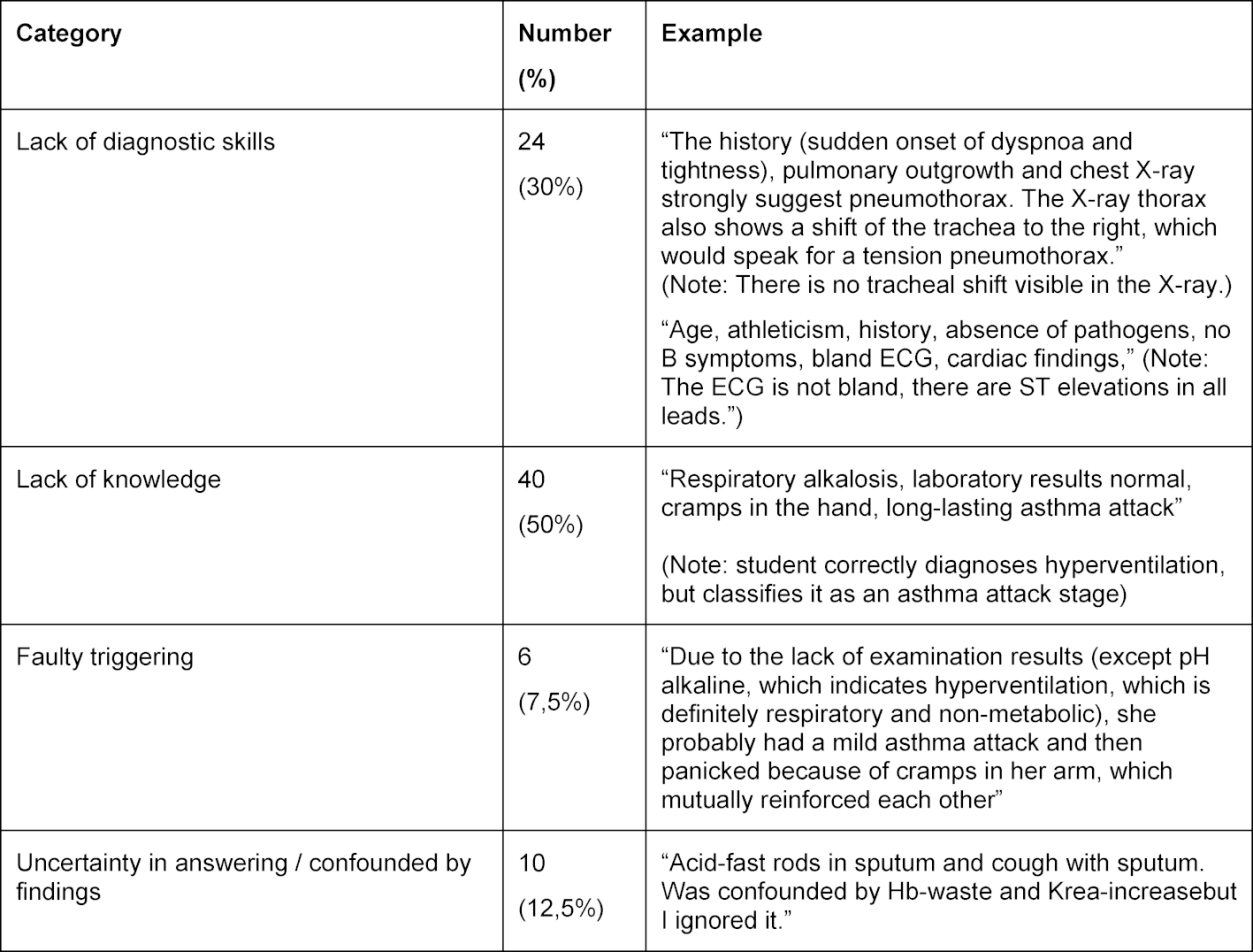
Categorization of wrong justifications
